# A Cell-Based Capture Assay for Rapid Virus Detection

**DOI:** 10.3390/v12101165

**Published:** 2020-10-15

**Authors:** Elad Milrot, Efi Makdasi, Boaz Politi, Tomer Israely, Orly Laskar

**Affiliations:** The Department of Infectious Diseases, Israel Institute for Biological Research, Ness-Ziona 74100, Israel; eladm@iibr.gov.il (E.M.); efim@iibr.gov.il (E.M.); boazp@iibr.gov.il (B.P.); tomeri@iibr.gov.il (T.I.)

**Keywords:** immuno-fluorescence assay, flow cytometry, West Nile Virus, Modified Vaccinia Ankara, Adenovirus, transmission electron microscopy

## Abstract

Routine methods for virus detection in clinical specimens rely on a variety of sensitive methods, such as genetic, cell culture and immuno-based assays. It is imperative that the detection assays would be reliable, reproducible, sensitive and rapid. Isolation of viruses from clinical samples is crucial for deeper virus identification and analysis. Here we introduce a rapid cell-based assay for isolation and detection of viruses. As a proof of concept several model viruses including West Nile Virus (WNV), Modified Vaccinia Ankara (MVA) and Adenovirus were chosen. Suspended Vero cells were employed to capture the viruses following specific antibody labeling which enables their detection by flow cytometry and immuno-fluorescence microscopy assays. Using flow cytometry, a dose response analysis was performed in which 3.6e4 pfu/mL and 1e6 pfu/mL of MVA and WNV could be detected within two hours, respectively. When spiked to commercial pooled human serum, detection sensitivity was slightly reduced to 3e6 pfu/mL for WNV, but remained essentially the same for MVA. In conclusion, the study demonstrates a robust and rapid methodology for virus detection using flow cytometry and fluorescence microscopy. We propose that this proof of concept may prove useful in identifying future pathogens.

## 1. Introduction

Viruses are intracellular obligate infectious parasites that rely on host machineries for replication and propagation. In the field of clinical virology, diagnosis of viruses relies on a variety of methods ranging from traditional methods, such as virus isolation in cell culture, serology and immuno-assays, to advanced sensitive techniques, such as qPCR, high throughput sequencing or microarray based approaches [1,2]. The significance of routine diagnosis is mainly in pathogen identification, surveillance, vaccine development and prevention and control of new outbreaks. Early diagnosis is crucial in order to facilitate rapid action to prevent further spread of the disease and allow risk assessment to be made, especially when an outbreak of an unknown pathogen occurs. Isolation of viruses from clinical specimens is vital for deeper analysis of virus identification and for vaccine development.

Most viruses are sized below the resolution limit of conventional microscopy and standard flow cytometry methods and fall within the range corresponding to optical, electrical, and filtered sheath buffer background noise [3]. Consequently, methods for detection of viruses mainly rely on replicating viruses in cells [4,5,6] or other methodologies such as bead based capture assays [7,8,9,10,11,12,13]. One main drawback of virus isolation and propagation in cell culture is that it is time consuming, possibly taking days or weeks for the virus to adjust and replicate. In addition, bead-based assays require prior knowledge about the pathogen therefore beads need to be conjugated to antibodies, calibrated, or activated (e.g., polymer coated beads), which make such methods time consuming and expensive. Polymer coated magnetic beads, such as polyethyleneimine (PEI), sulfonated magnetic beads (SO-magnetic), or poly(methyl vinyl ether-maleic anhydride), are widely used for virus capture and proved to be effective in concentrating diverse viruses for downstream detection assays, such as PCR, ELISA and immunoblotting [10,11,12,13,14,15,16].

However, a main drawback is that nonspecific binding of serum constituents hamper the ability of poly(methyl vinyl ether-maleic anhydride) beads to capture viruses [14] or high concentrations of fetal calf serum reduce the binding capability of PEI beads [12].

In this work, we present a novel, rapid assay for virus detection. The assay is based on virus capture by suspended Vero cells instead of beads. As in the case of beads, suspended cells expose their entire surface make them accessible for efficient virus adsorption. Vero cells present various receptors on their surface making them susceptible for binding by wide range of viruses. The detection of the captured viruses is carried out in subsequent assays including immuno-fluorescence microscopy assay (IFA) or fluorescence activated cell sorting (FACS). Several main advantages of the novel assay make it attractive for virus detection: 1. The assay is rapid as there are no required viral replication stages, thus direct and rapid virus detection is achieved. 2. The use of Vero cells is widely available in biological laboratories as they are permissive to a wide variety of viruses. 3. The assay is generic and can be set up for using various cell types for numerous viruses as needed. 4. The infectivity of the viruses retained after attachment to the cells.

## 2. Materials and Methods

### 2.1. Antibodies

FITC conjugated anti-Adenovirus antibodies were obtained from Abcam (Cat. ab87333). Goat anti-mouse FITC-conjugated IgG (Cat. F4018, Sigma, St. Louis, MO, USA) was used as a secondary antibody for WNV detection in IFA experiments. In-house production of rabbit anti-Vaccinia antibodies and mouse monoclonal anti-West Nile Virus antibodies were used in this study [17,18,19,20]. The antibodies were conjugated to Alexa 488 (Alexa Fluor 488 protein labeling kit, Cat. A10235Life Technologies, Grand Island, NY, USA) or FITC as described in the supplier’s protocols.

### 2.2. Cell Lines

Vero (CCL-81) and Vero E6 (CRL-1586) cell lines were obtained from the American Type Culture Collection (ATCC). Cells were cultured in Dulbecco’s modified Eagle’s medium (DMEM) containing 10% (*v*/*v*) fetal bovine serum (FBS), 1% L-glutamine, 1% (v/v) non-essential amino acid (NEAA) and 0.5% (*v*/*v*) antibiotics and incubated at 37 °C under 5% CO_2_ in a humidified incubator.

### 2.3. Viruses

Adenovirus (VR1504) was obtained from ATCC. The virus was propagated in Hela (ATCC) cells in MEM-Eagle Hank’s salts base medium (MEM) containing 2% (*v*/*v*) FBS, 1% L-glutamine, 1% (*v*/*v*) NEAA and 0.5% (*v*/*v*) antibiotics. When cytopathic effect was observed, the viruses were harvested and concentrated by ultracentrifugation as follows: The infected cells were sonicated and virus containing medium was layered on top of a 20% sucrose solution (containing 0.8% NaCl and 50 mM Tris-Hcl pH = 7.6) and then centrifuged 23,600 RPM for 2.5 h at 4 °C. After centrifugation, the supernatant was discarded and pellet containing viruses was added with fresh MEM medium. Titration of the viruses was carried out in Vero E6 cells as follows: Cells were seeded in 6-well plates (8 × 10^5^ cells/well) and left to adhere overnight. Serial dilutions of Adenovirus were prepared in MEM containing 2% FBS with NEAA, glutamine and antibiotics, and used to infect Vero E6 monolayers in duplicates (200 µL/well). Plates were incubated for 1 h at 37 °C to allow viral adsorption. Then, 3 mL/well of overlay (MEM containing 2% FBS and 0.4% Tragacanth (Merck, Rehovot, Israel) was added to each well and plates were further incubated at 37 °C, 5% CO_2_ for eight days. Growth media was then aspirated, and the cells were fixed and stained with crystal violet (Biological Industries, Beit Haemek, Israel) for 5 min at room temperature. The number of plaques in each well was determined.

West Nile Virus-82 (WNV) derived from plaque purification of a clinical isolate was previously characterized [21]. The virus was propagated on Vero cells and concentrated as previously described [22]. Modified Vaccinia Ankara (MVA) clonal isolate F6 at the 584th CEF passage were propagated and tittered as previously described [17,23]. The virus was propagated on secondary chicken embryo fibroblasts and titrated on BHK-21 cells.

### 2.4. Flow Cytometry Cell-based Capture Assay for Adenovirus, West Nile Virus and MVA

For the flow cytometry assays, Vero cells grown in 75cm flasks were harvested using trypsin (Cat. 03-051-5B, Beit Haemek, Beit Haemek, Israel) for several minutes. The cells were suspended in MEM solution containing 2% FBS (and supplements as described in Section 2.3) and enumerated using a hemocytometer. The cells were diluted to the appropriate concentration (as indicated in the Results section) and aliquoted in tubes, 250–300 µL cells/tube. Then, viruses spiked to either MEM or commercial pooled human serum (Cat. 2930149, MP Biomedicals, Irvine, CA, USA) or diluted serum were added (at the concentrations indicated in the results section) and incubated with the cells for one hour while shaking at 4C to allow attachment. After incubation, cells were centrifuged (7800× *g*, 5 min, 4 °C) and the supernatant was discarded. The cell pellet was then suspended in 30 µL of the relevant conjugated antibody diluted in PBS containing 1% BSA, 0.05% Tween-20 and incubated for 30–60 min. After labeling, cells were washed in 1ml PBS, then resuspended in PBS (300 µL) and analyzed by FACS.

### 2.5. Flow Cytometry Analysis

FACS analysis was carried out using BD LSRFortessa™ flow cytometer. Gating of Vero cells population was determined according to their size and granularity using forward scatter (FSC) and side-scatter (SSC) parameters. Quadrant settings were set according to the non-infected cells background fluorescence at Alexa488 channel. In these samples the background signal did not rise above 0.5% positive staining for MVA or 1% for WNV. The results were calculated as the percentage of positively stained Vero cells in the virus-exposed sample compared to the background (non-infected cells). The limit of detection was determined as three times the average value of the background signal from the non-infected cells.

### 2.6. Immuno-Fluorescence Microscopy Assays for the Detection of MVA and West Nile Virus after Capture with Vero Cells

The procedure of cell preparation for fluorescence microscopy analysis was carried out essentially as described in Section 2.4 with the following minor changes: after virus capture by Vero cells, the cells were centrifuged at 7800× *g* for 5 min to remove unbound viruses, and pellets (containing virus adsorbed cells) were re-suspended in 2%FBS-MEM and either seeded in Labtek dishes and incubated for 24 h or immediately processed for immuno-fluorescence microscopy; cells were spotted on glass slides and air dried. Fixation was carried out in cold 100% acetone for 20 min at −20 °C, followed by cell washing in Water. For MVA detection, Alexa 488 conjugated anti-vaccinia antibody diluted in 2% BSA, 0.02% Evans Blue was added for 30 min at 37 °C after which the cells were washed. For WNV detection, a two-step IFA was performed. Initially, a primary mouse monoclonal antibody diluted in 5% normal goat serum was added for 30 min at 37 °C. After antibody labeling the cells were washed and a secondary antibody conjugated to FITC diluted in 5% normal goat serum and 0.02% Evans Blue was added for 30 min. Finally, the slides were washed in water and then air dried. Visualization was carried out using an Axioscop 2 fluorescent microscope (Zeiss) equipped with a X40 magnification objective and a top mounted camera (DS-Fi3, Nikon). Images were recorded using NIS-Elements F4.6 software.

### 2.7. Negative Staining Electron Microscopy Analysis of MVA and Adenovirus after Capture with Vero Cells

For electron microscopy studies, Vero cells grown in 75 cm flasks were harvested using trypsin for several minutes. Cells were suspended in MEM solution containing 2% FBS (and supplements as described in Section 2.3), counted and diluted to 20,000 cells and aliquoted in separate tubes (300 µL per tube). Adenovirus and MVA were spiked to MEM containing 2% FBS, at final concentration of 1 × 10^6^ pfu/mL for MVA and 3 × 10^6^ pfu/mL for Adenovirus, respectively. The viruses were incubated with the cells for one hour at 4 °C with shaking. Cells were then centrifuged for 3 min at 7800× *g* to remove unbound viruses, and the supernatant was discarded. The cell pellets were re-suspended in 20 µL of 0.22 micron filtered DDW and sonicated for 10 min on ice. Roughly 10 µL of virus-containing lysate were incubated for 15 min on a 1 mg/mL Poly-L-Lysine (Cat. P2636, Sigma) treated carbon coated TEM grids (D1843-F, Ted Pella Inc. Redding, CA, USA). After sedimentation of the viruses, the grids were fixed in PBS containing 4% Paraformalehyde and 2.5% Glutaraldehyde for 15 min. The grids were then washed twice in filtered DDW and stained with 1% Phosphotungstic acid (PTA). After air-drying, samples were visualized in Tecnai T12 TEM (Thermo Fisher, OR, USA) operated at 120 kV equipped with a Gatan ES500W Erlangshen camera. 

### 2.8. Statistical Analysis

The data from the experiments conducted was analyzed using GraphPad Prism5 software. Results are expressed as mean ± standard error. Statistical significance was determined by two-tailed unpaired Student’s *t*-test. *p* value ≤ 0.05 was considered to be significant.

## 3. Results

### 3.1. Development of a Cell-based Immunoassay for the Detection of MVA, West Nile Virus and Adenovirus

**Figure 1:**
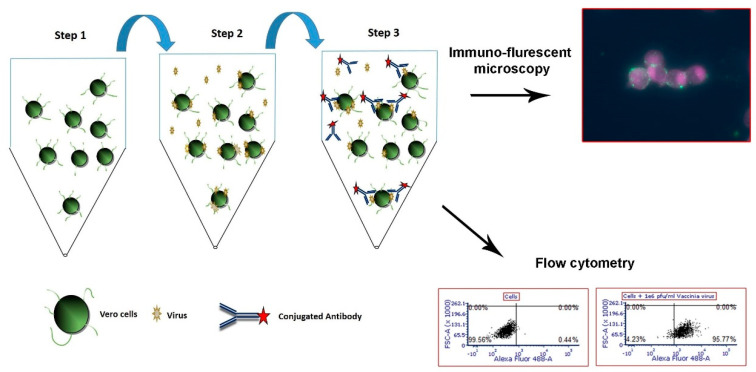
A schematic representation of the cell-based immuno-assay. Vero cells are harvested from cell culture flasks using trypsin (~15 min). The cells are then suspended in 2%FBS-MEM and aliquoted to tubes (step 1). The viruses are then added to the cells and incubated at 4 °C with shaking to allow adsorption to the cells surface (1 h) (step 2). After attachment to the cells, a specific fluorescent antibody is added (step 3, ~40 min) and the cells are analyzed in FACS or fluorescence microscopy.

In this study, Vero cells were used for non-specific capture of distinct viruses followed by specific detection based on antibodies. The basic principle of the assay developed in this work is depicted in Figure 1. Initially, detached Vero cells were suspended in infection medium and incubated with the virus-containing samples to allow viral attachment to the cells surface. Next, a fluorescently labeled antibody specific to the queried virus was added and the cells are imaged in a fluorescent microscope or analyzed by flow cytometry. The main advantage of the assay is that it extends for about 2 h, allowing a rapid detection of specific viruses.

**Figure 2:**
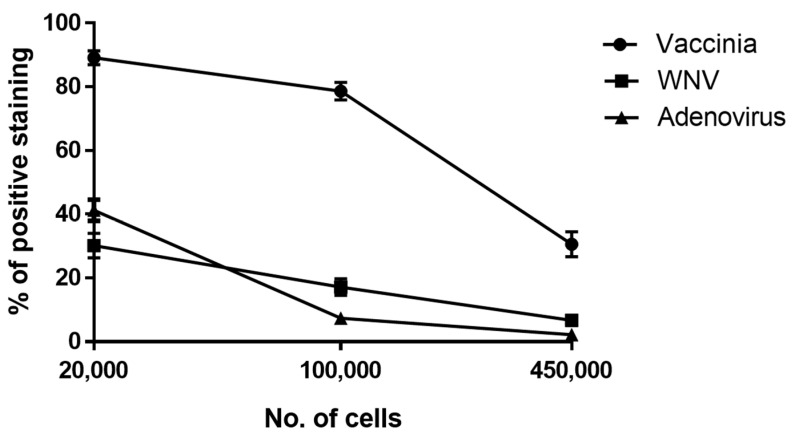
Optimization of cell number used for the cell-based immuno-assay in flow cytometry. The cells were harvested and diluted to achieve 20,000, 100,000 and 450,000 cells per tube. WNV were added at 9 × 10^6^ pfu/mL, MVA and Adenovirus were added at 1e6 pfu/mL for one hour with shaking at 4 °C to allow attachment. The cells were then immuno-labeled with the relevant fluorescent antibody and analyzed in FACS. The results shown represent average values of positive staining ± SEM from 3 independent experiments in biological duplicates for each of the viruses.

As an initial proof of concept, we tested this methodology for its ability to detect three different viruses: Modified Vaccinia Ankara (MVA), West Nile Virus (WNV) and Adenovirus. These viruses were chosen as they infect Vero cells and represent RNA (WNV) and DNA viruses (MVA and Adenovirus) from three different families (*Flaviviridae*, *Poxviridae* and *Adenoviridae*, respectively). We first performed a calibration assay to determine the optimal number of cells for reaching the highest fluorescent signal in FACS. This was defined as achieving the highest proportion of positively stained cells out of the tested population. For that purpose, we used a specific and constant virus concentration (1 × 10^6^ pfu/mL for MVA and Adenovirus or 9 × 10^6^ pfu/mL for WNV) along with several cell concentrations. The percentage of virus-positive cells (cells that adsorbed to virions) was determined for each of the viruses relative to control (uninfected cells). As shown in Figure 2, the highest signal acquired for MVA, WNV and Adenovirus was obtained using 20,000 cells per tube and the signal was gradually decreased as the number of cells per tube was elevated. Thus, for subsequent experiments, 20,000 Vero cells per tube were used.

**Figure 3:**
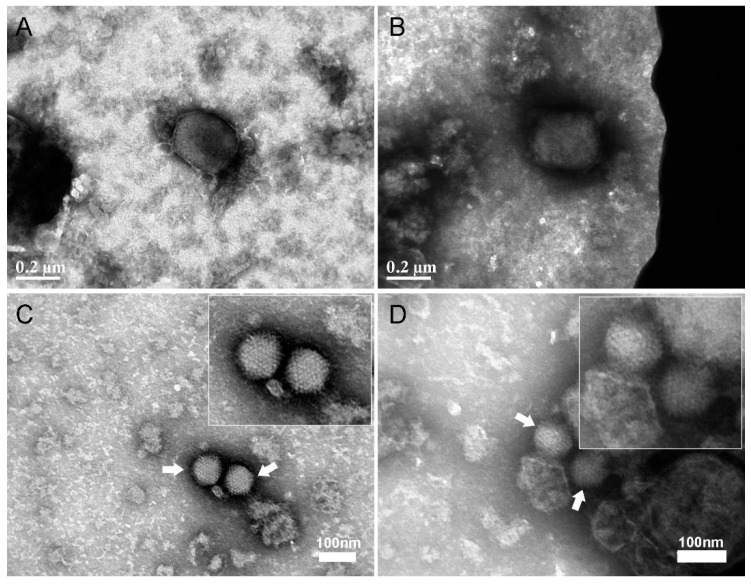
Negative staining TEM imaging of MVA and Adenovirus after capture and release from Vero cells. MVA and Adenovirus spiked in 2% FBS-MEM were added to Vero cells for one hour to allow attachment. After one hour, the cells (containing adsorbed viruses) were pelleted and then sonicated to release the bound viruses. The viruses were then added to TEM girds, fixed and stained with 1% PTA. (**A**,**B**) The traditional “brick” shape structure of MVA was seen among cell debris. (**C**,**D**) The icosahedral Adenovirus particles could be observed among cell debris. Representative images are from two experiment in biological duplicates. Insets shows high magnification of the viruses marked with two white arrows in panel (**C**,**D**).

One of the queries needing to be considered is whether the positively stained cells observed in the flow cytometry analysis adsorbed complete virus particles. To this end, we performed TEM analysis of negatively stained Adenoviruses and MVA after capture with (20,000) Vero cells. These viruses were chosen as they can be easily recognized in TEM according to their unique conspicuous morphologies. MVA exhibit a “brick” structure at sizes ranging from 300–350 nm X 140–260 nm and Adenovirus is a ~80 nm icosahedral shaped virus [24,25]. In the TEM, intact MVA and Adenovirus virions were seen dispersed among cell debris (Figure 3).

As we encountered technical difficulties with the propagation of Adenovirus to high titers, from this point comprehensive studies were conducted on MVA and WNV.

**Figure 4:**
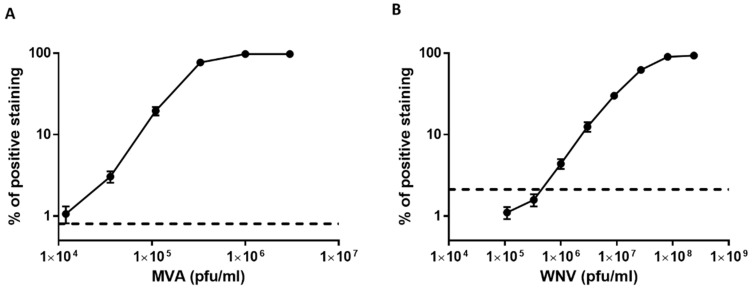
Dose response analysis of MVA and West Nile Virus after capture by Vero cells. (**A**,**B**) The cells were harvested and diluted to achieve 20,000 cells per tube. WNV and MVA (diluted in 2% FBS-MEM) were added at the indicated concentrations for one hour with shaking at 4 °C to allow attachment. The cells were then immuno-labeled with a relevant fluorescent antibody and analyzed by FACS. Dashed black line represents the limit of detection of the assay (2.12% of positive staining for WNV and 0.8% for MVA) which was determined by 3 times the mean value of background noise from non-infected cells. The results shown represent average values ± SEM from 3 independent experiments in biological duplicates for each of the viruses.

Initially, we sought to determine the dynamic range and sensitivity of the assay. For this purpose, a dose response assay was performed in flow cytometry using MVA and WNV (Figure 4). A threefold serial dilution of the virus stocks was applied to these experiments and 20,000 Vero cells were used. The percentage of positively labeled cells was proportional to the virus concentrations up to 1 × 10^6^ pfu/mL and 8.1 × 10^7^ pfu/mL for MVA and WNV, respectively. At virus concentrations of 1 × 10^6^ pfu/mL (MVA) and 1 × 10^8^ pfu/mL (WNV), all cells were positively labeled. The results demonstrate a wide dynamic range with a detection threshold of 3.6 × 10^4^ pfu/mL and 1 × 10^6^ pfu/mL for MVA and WNV, respectively. The limit of detection (dashed black bar) of the assay was determined by 3 times the mean value of background noise from non-infected cells (see Materials and Methods).

**Figure 5:**
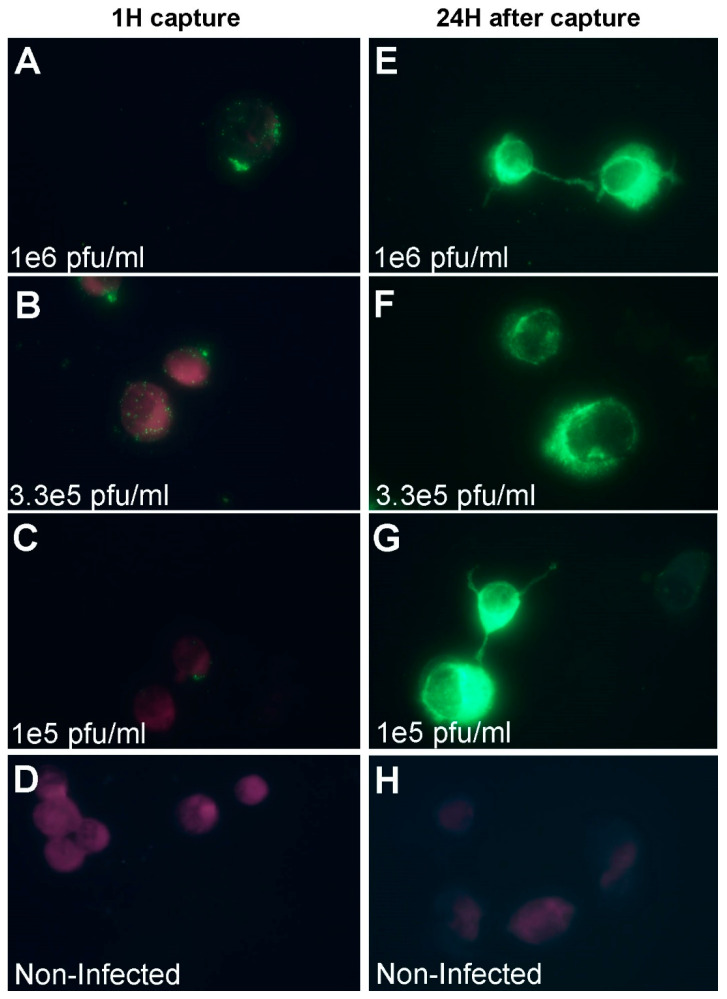
Fluorescence microscopy imaging of MVA after capture with Vero cells. (**A**–**D**) Suspended Vero cells were mixed with MVA at the indicated concentrations for one hour with shaking to allow attachment. After capture, the cells were pelleted and processed directly to IFA. The cells are labeled with purple color and viruses are in green. (**A**,**B**) Numerous viral particles could be observed on the cells surface after an hour of incubation with the cells. (**C**) Few MVA particles were seen on the cells surface. (**D**) No virus bound cells were observed in the control cells. (**E**–**G**) Suspended Vero cells were mixed with MVA at the indicated concentrations for one hour with shaking at 4 °C to allow attachment and then seeded in Labtek dishes for additional 24 h to allow virus replication. The cells were then processed to IFA. Intensive cellular staining was noted at all virus doses. (**H**) No cellular staining was observed in non-infected cells after 24 h of incubation.

As mentioned above, IFA is traditionally used for virus detection, usually after virus replication which might take several days. In the current experiment, we compared the sensitivity of the rapid IFA (after one hour of capture with Vero cells) and the traditional IFA (after 24 h of incubation). For these experiments, suspended Vero cells were incubated with MVA and after virus capture the cells were either processed directly to immuno-fluorescence microscopy, or seeded in Labtek dishes and incubated for additional 24 h to allow virus replication. The results from the direct IFA show a dose dependent relation between the virus dose and the staining intensity (Figure 5A–C). Numerous virus particles (green spots) were visualized on the cells surface (the cells are denoted by purple color) when MVA was added at 1 × 10^6^ pfu/mL or 3.3 × 10^5^ pfu/mL (Figure 5A,B). At lower virus concentrations (1 × 10^5^ pfu/mL) low amounts of virus particles could be observed in only a small fraction of the cells (Figure 5C). In contrast, at 24 h post virus capture, almost the entire cells area was intensively labeled at all virus doses, indicating a massive virus replication in the cells (Figure 5E–G). Non-infected cells did not exhibit any labeling when either processed directly or after 24 h (Figure 5D,H). Notably, the intensity of cellular staining (from Evan’s blue reagent) varied between cells in the same sample. This phenomenon is most probably due to variance in penetration ability of the stain to the cells.

**Figure 6:**
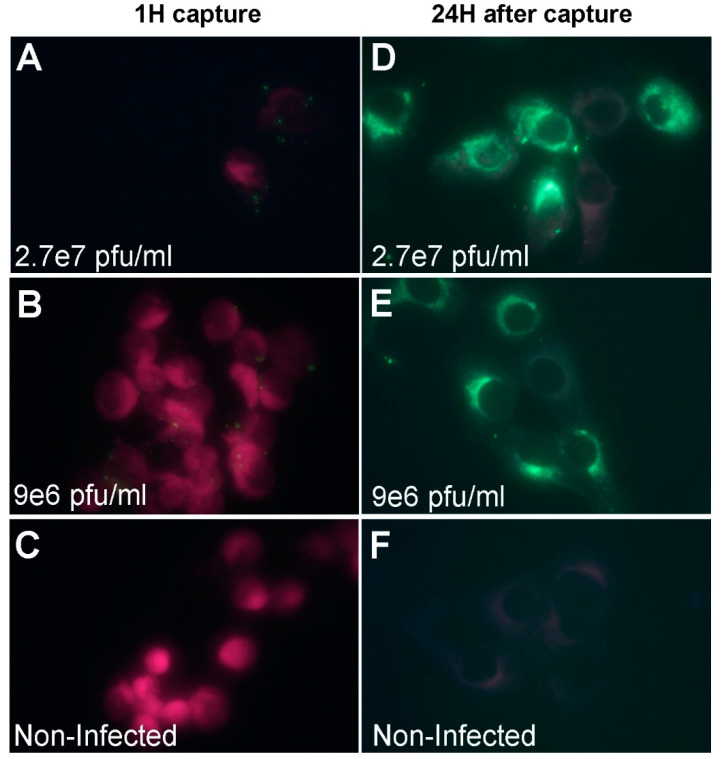
Fluorescence microscopy imaging of WNV after capture with Vero cells. (**A**–**C**) Suspended Vero cells were mixed with WNV at the indicated concentrations for one hour with shaking to allow attachment. After capture, the cells were pelleted and processed to IFA. Cells were labeled with purple color and viruses are in green. (**A**,**B**) Multiple viral particles could be observed on the cells surface. (**C**) Non-infected cells did not show any staining. (**D**,**E**) Suspended Vero cells were mixed with WNV at the indicated concentrations for one hour with shaking to allow attachment. After capture the cells were pelleted and seeded in Labtek dishes for additional 24 h to allow virus replication. Intensive cellular staining was noted at 2.7 × 10^7^ pfu/mL and 9 × 10^6^ pfu/mL virus concentrations. (**F**) No cellular staining was observed in non-infected cells after 24 h of incubation.

In a similar manner, the IFA experiments were conducted on WNV. Representative images are shown in Figure 6. Multiple virus particles (green spots) were seen on the cells surface (the cells are denoted by purple color) when WNV was added at 2.7 × 10^7^ pfu/mL or 9 × 10^6^ pfu/mL (Figure 6A,B). In contrast, at 24 h post virus capture, the staining intensity was elevated and the pattern was changed from single sparse dots to intensive spread inside the cells, indicating virus replication (Figure 6D,E). Non-infected cells did not exhibit any labeling when either processed directly or after 24 h (Figure 6C,F).

### 3.2. Adaptation of the Cell-Based Assay to Human Serum Samples

**Figure 7:**
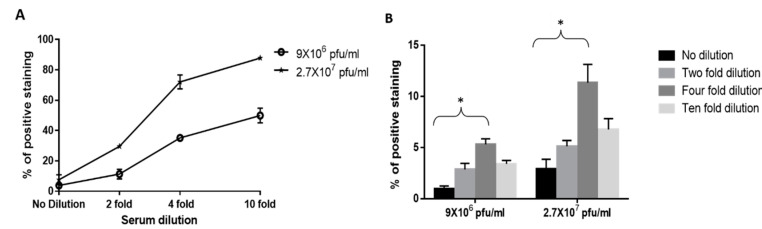
Adaption of the capture assay to commercial human serum. (**A**) Cells were harvested and diluted to achieve 20,000 cells per tube. WNV was spiked at the indicated concentrations into human serum or diluted serum. Next, the viruses were added to Vero cells and incubated for one hour with shaking at 4 °C to allow attachment. The cells were then immuno-labeled with anti-WNV:FITC antibody and analyzed by FACS. The results shown are average values of positive staining ± SEM from one experiment in biological duplicates. (**B**) WNV was spiked at 9 × 10^6^ pfu/mL and 2.7 × 10^7^ pfu/mL into commercial human serum. After spiking, the serum containing virus was diluted with PBS and then added to the cells for one hour with shaking at 4 °C to allow attachment. The cells were then immuno-labeled with anti-WNV:FITC antibody and analyzed by FACS. The results shown are from two experiments in biological duplicates. (*) Statistical significance was determined by 2 tailed unpaired student’s *t*-test. *p* value ≤ 0.05 was considered to be significant.

Isolation of viruses from clinical samples is challenging but important for virus identification. In practice, viruses-containing clinical samples, such as serum contain high protein quantities, such as serum albumin and antibodies. Therefore, for cell-based assays, such as plaque assay, serum dilutions are conducted in order to reduce the inhibitory effects of the serum. We therefore set out to investigate the effect of human serum on the capture of WNV and MVA by Vero cells. Initially, we tested the inhibitory effect of a commercial pooled human serum product on the capture of WNV. For this assay, the virus was initially spiked at two constant concentrations (9 × 10^6^ pfu/mL and 2.7 × 10^7^ pfu/mL) to serum and serum dilutions and only then added to Vero cells. Following the capture process, cells were immuno-labeled with anti-WNV antibodies and analyzed by FACS. As can be seen in Figure 7A, serum has an inhibitory effect on virus binding, though binding ability can be recovered by using diluted serum. For instance, when WNV was spiked to non-diluted serum at 2.7 × 10^7^ pfu/mL or 9 × 10^6^ pfu/mL there were only ~4% positively stained cells. When WNV was spiked at 9 × 10^6^ pfu/mL and 2.7 × 10^7^ pfu/mL to two-fold diluted serum there was a slight elevation in staining efficiency reaching 11% and 29%, respectively. When WNV was spiked at 9 × 10^6^ pfu/mL and 2.7 × 10^7^ pfu/mL to tenfold serum dilutions, a dramatic increase in the staining efficiency was observed reaching 50% and 87% of positively stained cells, respectively (Figure 7A).

The results of this experiment show that human serum has an inhibitory effect on the binding of the viruses to the cells and that this inhibition was partly alleviated by dilution. At this point, we sought to determine the serum dilution at which we get the highest positive staining of the cells, in the FACS assay. For this purpose, we spiked two constant WNV concentrations to human serum (9 × 10^6^ pfu/mL and 2.7 × 10^7^ pfu/mL) and then diluted the serum. Practically, in the context of the current experiment and in contrast to the previous experiment (shown in Figure 7A), the dilution in this case is of the serum containing viruses; thus, after dilution, the virus concentration is reduced. The diluted and undiluted samples were then added to Vero cells and after capture, the cells were immuno-labeled and analyzed by FACS. Although the percentages of the positive cells were minimal at all serum treatments, the trends from this graph are clear. Two fold dilution is not enough to overcome the inhibitory effect of the serum while four-fold dilution resulted a significant increase (*p* = 0.0007 for 9 × 10^6^ pfu/mL, *p* = 0.0061 for 2.7 × 10^7^ pfu/mL) in the percentage of positive cell population (Figure 7B). Tenfold dilution of the serum sample resulted a reduced virus concentration yielding low positive staining compared to four-fold dilution. Hence, for our subsequent experiments we used four-fold serum dilution.

### 3.3. Dose Response Analysis of MVA and West Nile Virus Spiked into Human Serum

**Figure 8:**
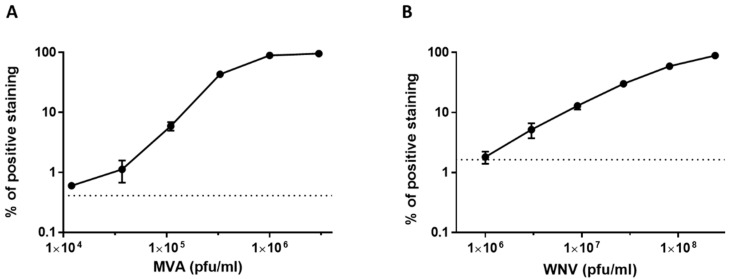
Dose response analysis of MVA and West Nile virus spiked to fourfold-diluted human serum after capture with Vero cells. (**A**,**B**) The cells were harvested and diluted to achieve 20,000 cells per tube. Next, WNV or MVA spiked to fourfold diluted human serum were added to the cells and incubated for one hour with shaking at 4 °C to allow attachment. The cells were then immuno-labeled with a relevant fluorescent antibody and analyzed by FACS. The results shown are average values of positive staining ± SEM from 3 and 4 experiments in biological duplicates for MVA and WNV, respectively.

In order to evaluate the sensitivity of detection in serum samples we performed dose response analysis of MVA and WNV spiked into four-fold diluted serum. A threefold serial dilution of the virus stocks was applied to these experiments and 20,000 Vero cells were used. As shown in Figure 8, detection of MVA and WNV was achieved at concentrations of 3.6 × 10^4^ pfu/mL and 3 × 10^6^ pfu/mL, respectively. As the virus concentration increased, the fraction of positive cells increased correspondingly, peaking at 1 × 10^6^ pfu/mL for MVA and 2.4 × 10^8^ pfu/mL for WNV. The results obtained for MVA were comparable to those shown in Figure 4. In the case of WNV, there was a slight reduction in the sensitivity of the assay with the serum samples compared to infection medium (Figure 4).

## 4. Discussion

The current study describes a novel rapid cell-based assay for virus detection. While demonstrated a proof of concept mainly using three model viruses (WNV, MVA and Adenovirus), conceivably it could be broadened to a wide range of additional viruses.

From our initial flow cytometry experiments we determined the number of cells needed to achieve the highest fraction of cells that are positively stained. As the aim of the study was to develop a virus detection assay, we were interested in achieving the highest signal, thus we chose to work with a low number of cells bearing in mind that we might “miss” part of the viruses that are not adsorbed to the cells. Most probably, lower amounts of cells might yield higher signal, however in our experience it is technically difficult to maintain a low number of cells, during preparation. Theoretically, a high number of cells (100,000 or 450,000) should have resulted in an increase of adsorbed viruses to the cells as there are more available receptors. In practice, in the flow cytometry analysis, a decreased positive staining of the cells was noted as the number of cells increased. Most probably at low cell number (20,000), a high number of virus particles attach individual cells, resulting a shift of the positively stained population; however, when higher amount of cell are applied (100,000 or 450,000), the viruses spread among many more cells, resulting lower positive staining of the cells population, as individual cells catch less viruses. This assumption is further corroborated by the IFA analysis, which showed a dose dependent virus staining of the cells (Figure 5 and Figure 6).

Generally, traditional IFA experiments are carried out following virus replication in confluent cells, a process which extend several days. Notably, IFA assays for the detection of WNV in clinical specimens were already demonstrated before. An example of such an assay can be found in [26]. In this study, WNV exposure was monitored by using WNV infected Vero cells fixed on slides and detection by serum containing specific anti-WNV IgG and IgM antibodies. This type of immuno-based serological assay aims to detect previous exposure to WNV, conceptually demonstrates different approach of virus detection, rather than direct viral detection as demonstrated in the current work. The results of the IFA presented in this work show that the sensitivity of the capture assay is enhanced after 24 h of virus incubation with the cells. The reduction of the incubation time to 1 h and the low number of cells used for the capture assay enabled us to perform a rapid IFA but with the disadvantage of reduction in sensitivity. However, for rapid virus detection, for instance, at an emergent situation when a detection of a suspected virus is needed, 1-h incubation with the cells is also valuable and applicable.

Isolation of viable viruses is important for virus characterization and vaccine development; however, conventional assays do not permit isolation of infectious particles because the viruses are inactivated during sample preparation, for instance ELISA, western blotting or PCR. The method described herein revealed as an effective approach for rapid capturing of intact infective virus particles from single samples which is crucial for downstream virolgical applications, such as virus titration and enrichment.

Typically, cell-based immuno-assays for viruses in FACS focus on detection, quantification and titration, hence extend several days in order to allow virus replication in the cells [4,5,6,27,28]. As, in the current study, we were interested in a rapid virus detection assay, we performed a FACS analysis after 1 h of virus incubation with the cells, which is a reasonable time frame for virus attachment. In addition, the assays were performed at 4 °C and without cellular perforation steps in order to improve the detection of the viruses adsorbed to cells surface. Thus, we suggest that the flow cytometer analyses viruses adsorbed to the cells surface, a conjecture that is further supported by the immuno-florescent microscopy assays (Figure 5 and Figure 6).

The flow cytometry dose response analyses demonstrate that we were able to detect viruses at a wide range of virus concentrations for both WNV and MVA. At permissive conditions (virus spiked into infection medium), virus concentrations as low as 3.6 × 10^4^ pfu/mL for MVA or 1 × 10^6^ pfu/mL for WNV were detected. Previous studies showed that the sensitivity of detection can be enhanced at long incubation periods after virus replication and enrichment in cells [5,6]. The scope of the current work covers a comprehensive study that describes a rapid and general method for virus capture and detection that is independent on prior replication stages of the virus or viral genomes; consequently, we “pay” by reduced sensitivity, compared to other methods, such as ELISA, which requires prior knowledge and specific reagents to achieve its high sensitivity. Albeit as low as ~60 pfu/mL or above 1 × 10^3^ pfu/mL can be detected in ELISA for WNV and orthopox-viruses, respectively [29,30], a comparative study of our method to other methods is beyond the scope of this study and might be investigated in the future. There are several factors that crucially affect the sensitivity of the virus capture flow cytometry assay:

1. Antibody type, concentration, specificity and conjugation. As the assay strongly depends on reagents such as antibodies, the sensitivity may change accordingly.

2. The number of cells used for virus capture (Figure 1).

3. Virus type and receptor copy number on the host cells membranes. Theoretically, the higher the copy number of the virus receptors the higher is the capture ability of the cells along with the sensitivity of the assay. Thus, with this line of thought we used suspended cells to make sure that entire cell surface (and thus receptors) is accessible for the viruses.

These factors can explain the results presented in Figure 2 showing the variance in positive staining between the three different viruses.

Previous studies showed that serum eliminate virus binding ability of anionic coated beads [14] and that high concentrations of fetal calf serum adsorb non-specifically to PEI beads reducing their virus binding ability [12]. In order to investigate whether the detection assay can be performed with serum, we used commercial pooled human serum for virus spiking and performed FACS analysis of the cells after incubation with the viruses. The analyses showed that serum has inhibitory effects on virus binding. This limitation was overcome by serum dilution. The standard curves obtained for viruses that were spiked to diluted serum were comparable to those acquired with permissive conditions, with a slight reduction in sensitivity for WNV. Thus, serum dilution is an efficient way to preserve the virus binding ability of the cells.

The applicability of the cell-based assay described here to clinical specimens such as serum make it attractive to be applied in an emergent case when a specific virus detection is needed. Further experiments should be considered in order to confirm the applicability of the assay to other clinical specimens such as CSF or other body fluids.

The use of cell lines for virus capture in comparison to beads has several advantages: 1. Vero are easy to maintain and widely used in biological laboratories as they are permissive to a wide variety of viruses. The queried virus determines the cells needed for propagation [4,5]. 2. Virus capture with suspended Vero cells preserves virus morphology and infectivity, which are needed for further analyses.

Early detection and isolation of viruses are both important for disease spreading and facilitating rapid prevention actions. The current study presents a proof of concept of a cell-based capture assay for virus detection. The assay is rapid, flexible and can be broadened according to other “suspected” query virus. This assay can be utilized to diagnostic laboratories for the detection of known local or emerging viral threats and help to prevent disease spreading in the community.
